# Retroperitoneal hematoma from lumbar artery as an unusual complication after rectal perforation with massive retroperitoneal emphysema

**DOI:** 10.1093/jscr/rjy332

**Published:** 2018-12-12

**Authors:** Takashi Miyata, Yuta Fujiwara, Koji Nishijima, Fumio Futagami, Takashi Nakamura, Takahisa Yamaguchi, Koichi Okamoto, Isamu Makino, Tomoharu Miyashita, Tetsuo Ohta

**Affiliations:** 1 Department of Surgery, Japanese Red Cross Kanazawa Hospital, Kanazawa, Ishikawa 9218162, Japan; 2 Department of Gastroenterological Surgery, Division of Cancer Medicine Graduate School of Medicine, Kanazawa University, Kanazawa, Ishikawa, Japan

## Abstract

The patient was a 54-year-old woman diagnosed with adult Still’s disease, undergoing high-dose steroid and immunosuppressant therapy for 5 years, who was admitted to our hospital with abdominal pain. Computed tomography (CT) revealed pneumoperitoneum around the rectum and a large quantity of retroperitonal emphysema around the inferior vena cava, aorta and left kidney. An emergency laparotomy was performed. Intraoperative observation revealed a perforation on the mesenteric side of the rectum due to diverticulum, and Hartmann’s operation was performed. Deep tenderness and anemia were observed 4 days postoperatively. CT revealed extravasation in the left retroperitoneal space and a retroperitoneal hematoma, and emergency embolization of lumbar arteries was performed. Retroperitoneal bleeding associated with peritonitis after surgery is very rare. We surmised that higher-dose immunosuppressive therapy and gastrointestinal perforation with emphysema in the retroperitoneum induced lumbar artery bleeding. Clinicians should consider these factors as a potential cause of retroperitoneal hematoma.

## INTRODUCTION

Retroperitoneal hematoma is a rare clinical entity that can be fatal and requires rapid treatment for patient survival [1]. It is frequently seen with a complication such as femoral artery catheterization, pelvic or lumbar trauma, rupture of aneurysms, or anticoagulation therapy [2, 3]. However, retroperitoneal hematoma caused by postoperative peritonitis with retroperitoneal emphysema is extremely rare. We herein report the case of spontaneous retroperitoneal hematoma occurring in a woman with adult Still’s disease undergoing high-dose steroid and cyclosporine therapy who was precisely diagnosed and successfully treated.

## CASE REPORT

**Figure 1:**
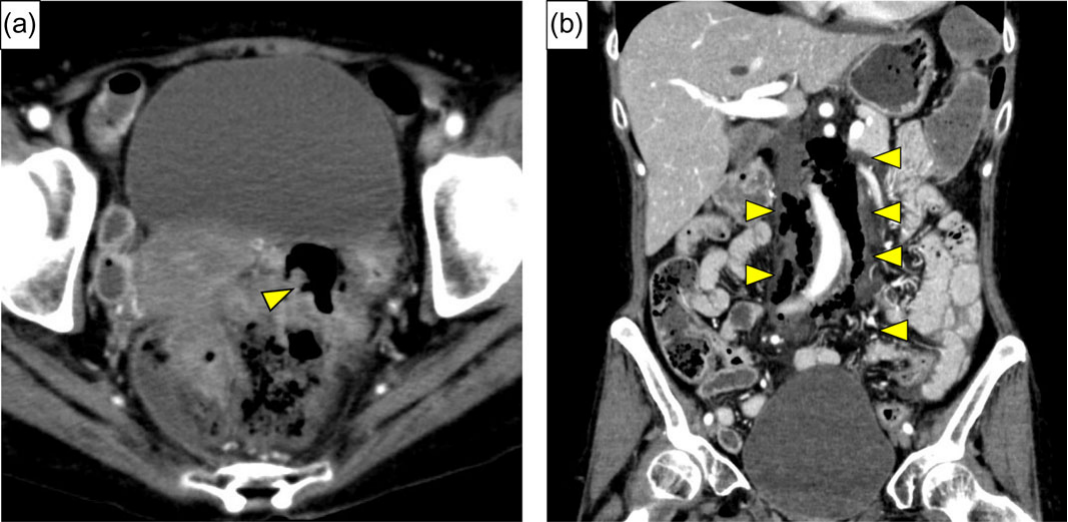
(**a**) Enhanced abdominal CT showed free air around the rectum (yellow arrow), and strongly suggested perforation of the rectum. (**b**) Massive continuous pneumoretroperitoneum from rectum was revealed (yellow arrows).

**Figure 2:**
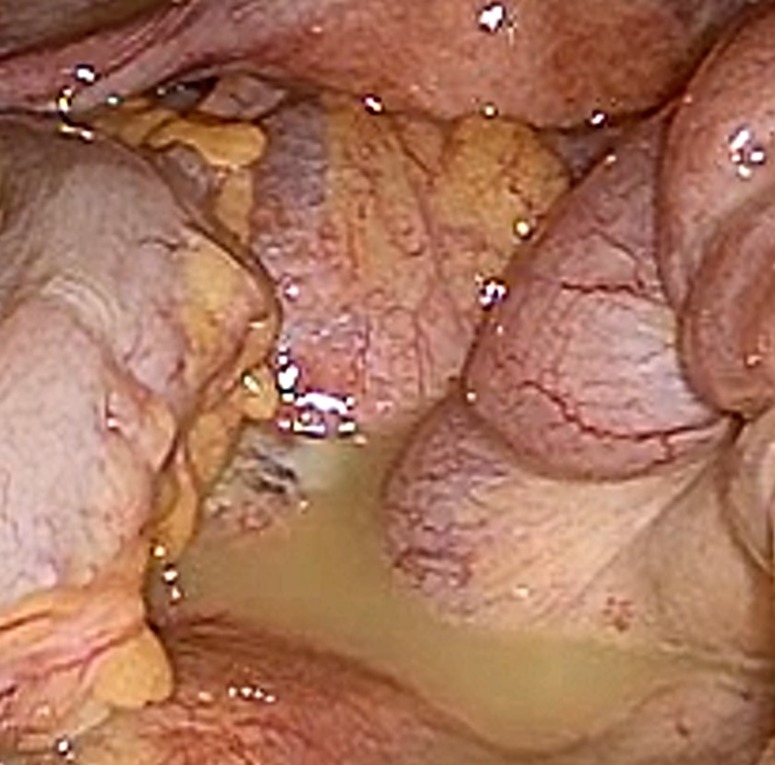
Laparotomy revealed the peritoneal fluid collection.

**Figure 3:**
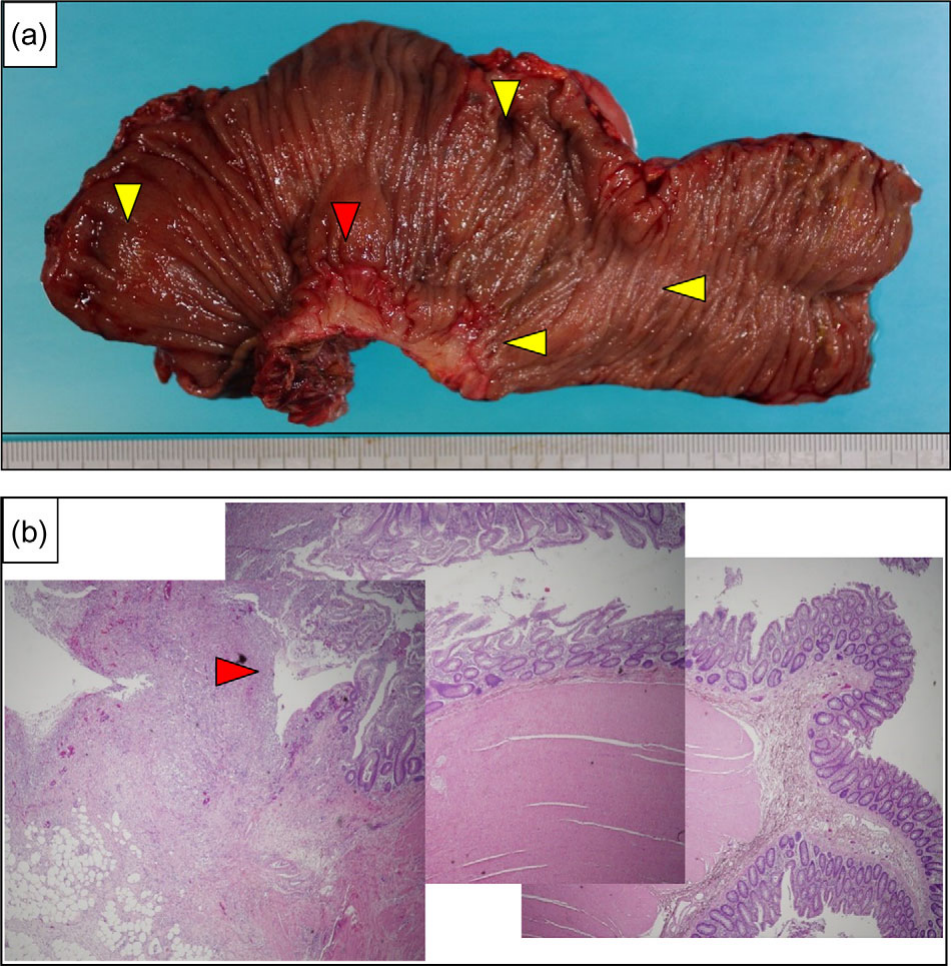
(**a**) The resected specimen showed perforation at the rectum (red arrow), and diverticulitis was apparent (yellow arrows). (**b**) A photomicrograph showed diverticulum perforation over the posterior wall of the rectum (red arrow).

**Figure 4:**
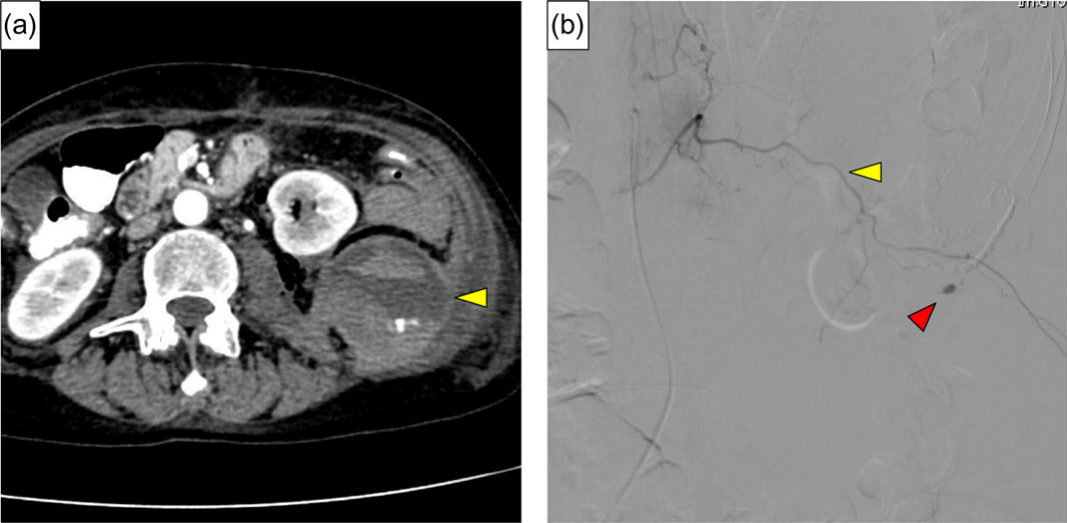
(**a**) Contrast extravasation from lumbar artery and hematoma expansion lifting the left kidney was identified (yellow arrow). (**b**) Angiography revealed extravasation of contrast medium (red arrow) from the left first lumbar artery (yellow arrow).

**Figure 5:**
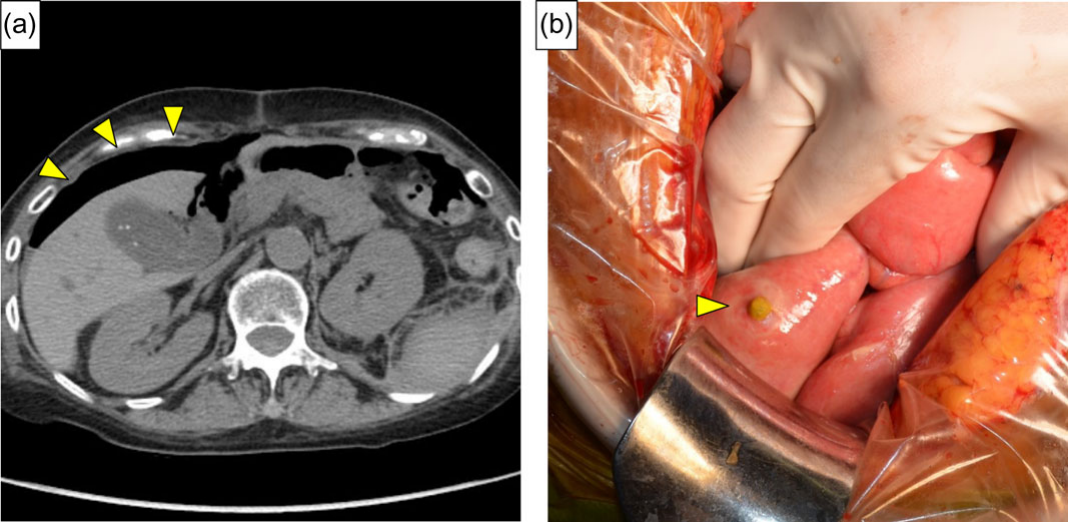
(**a**) CT showed free air around the right lateral region (yellow arrows). (**b**) Intraoperative findings showed perforation of the cecum with no obvious masses (yellow arrow).

A 54-year-old woman was referred to our hospital because of abdominal pain. She had a history of adult Still’s disease at age 49 and underwent treatment with oral prednisolone, 90 mg/day and cyclosporine, 175 mg/day. A physical examination revealed deep tenderness in the abdomen. Laboratory data showed slight leukocytosis (white blood cell count 9100/μL) with a moderately elevated C-reactive protein level (9.3 mg/dL), while other data, including blood coagulation factor, were within normal ranges. Computed tomography (CT) revealed a small amount of extra-intestinal free air around the rectum and massive retroperitoneal emphysema between the rectum and the left kidney (Fig. 1a and b). Arterial aneurysm was not confirmed. Based on a preoperative diagnosis of rectal perforation, emergency laparotomy was performed, which confirmed peritoneal fluid collection (Fig. 2) and rectum perforation on the retroperitoneal side. After aspiration of the pus and irrigation of the area with saline, Hartmann’s operation was performed (Fig. 3a). Although she was undergoing immunosuppressive treatment, pathological study disclosed no association between diverticulum perforation and cytomegalovirus enteritis (Fig. 3b). On postoperative Day 4, she suffered a sudden intolerable left flank pain; her hemoglobin level was 7.5 g/dL, and slight prolongation of prothrombin time was recognized. CT revealed a left retroperitoneal hematoma and extravasation from the left first lumbar arteries (Fig. 4a). Emergency transarterial angiography and lumbar artery embolization was performed (Fig. 4b). On Day 20 after the first operation the patient felt a sudden right flank pain, and CT confirmed intra-abdominal free air (Fig. 5a). A second emergency laparotomy was performed, which revealed cecal perforation with no obvious masses (Fig. 5b). Perforation resulting from diverticulum was suspected, and an ileostomy without intraperitoneal anastomosis was performed because of concern about anastomotic leakage. Postoperatively the patient developed an intra-abdominal abscess, surgical site infection (Clavien-Dindo IIIa) and pneumonia (Clavien-Dindo II), which were treated conservatively. Although it took time to rehabilitate the patient and control the adult Still’s disease, she was discharged on Day 212 after the first operation. The patient is now doing well with comfortable activity of daily life.

## DISCUSSION

Retroperitoneal hematoma is a well-known, but rare, clinical condition that can be potentially lethal [1]. It is most frequently seen as a complication of interventions such as femoral artery catheterizations and pelvic or lumbar trauma [2]. In this report, we present an extremely rare case of retroperitoneal hematoma after surgery of rectal perforation with peritonitis. Two clinical circumstances, pneumoretroperitoneum and immunosuppressive therapy, were potential etiologic factors leading to retroperitoneal hematoma.

Pneumoretroperitoneum caused by colonic retroperitoneal perforation is a rare clinical condition, the most common cause of which is diverticular disease [4]. Colonic retroperitoneal perforation leads to the spread of abscess, gas and necrosis along the retroperitoneum, occasionally extending to the mediastinum. The proposed surgical procedure for this condition is colonic resection and radical debridement [4]. To the best of our knowledge, however, there have been no reports in the English literature of the postoperative complication of retroperitoneal hematoma associated with pneumoretroperitoneum caused by colonic retroperitoneal perforation.

The association between steroid use and gastrointestinal ulceration or perforation is now well established. Chronic high-dose steroid treatment has been related to decline of some cell-mediated lymphocyte functions, suppression of bactericidal activities of neutrophils and fragility of tissue [5]. The present patient, who had adult Still’s disease, had been taking high-dose steroids for a long time, and gastrointestinal perforation occurred twice in a short period. Although the immunosuppressive therapy must be considered as potentially inducing the event, thus far there have been no reports of retroperitoneal hematoma associated with immunosuppressive therapy for adult Still’s disease.

In general, the pathophysiology of spontaneous retroperitoneal bleeding is yet to be determined. It has been hypothesized that diffused occult vasculopathy and minor trauma in the microcirculation in the retroperitoneum may give rise to fragility and proneness to rupture [6]. Others have suggested that anticoagulation-induced immune microangiopathy may be responsible [7]. In this case, drainage of the abscess in the retroperitoneal space, vascular tissue weakness caused by chronic high-dose immunosuppressants, and anticoagulation resulting from peritonitis were considered the inciting factors leading to spontaneous retroperitoneal bleeds.

In conclusion, despite not finding any obvious cause of bleeding, we experienced an extremely rare case of retroperitoneal hematoma after surgery for colonic perforation of the rectum with retroperitoneal emphysema in a woman undergoing chronic high-dose immunosuppressive therapy for adult Still’s disease. Retroperitoneal bleeding should be suspected, and early treatment may be essential for improved prognosis when patients receiving a higher dose of immunosuppressants present with abdominal pain.
